# EGFR Family Members’ Regulation of Autophagy Is at a Crossroads of Cell Survival and Death in Cancer

**DOI:** 10.3390/cancers9040027

**Published:** 2017-03-24

**Authors:** Elizabeth Henson, Yongqiang Chen, Spencer Gibson

**Affiliations:** 1 Research Institute in Oncology and Hematology, CancerCare Manitoba, 675 McDermot Ave., Winnipeg, MB R3E 0V9, Canada; Elizabeth.Henson@umanitoba.ca (E.H.); yongqiangchen2007@gmail.com (Y.C.); 2 Department of Biochemistry and Medical Genetics, Faculty of Health Sciences, University of Manitoba, Winnipeg, MB R3E 0V9, Canada

**Keywords:** epidermal growth factor, autophagy, signaling, mitochondria, beclin-1

## Abstract

The epidermal growth factor receptor (EGFR) signaling pathways are altered in many cancers contributing to increased cell survival. These alterations are caused mainly through increased expression or mutation of EGFR family members EGFR, ErbB2, ErbB3, and ErbB4. These receptors have been successfully targeted for cancer therapy. Specifically, a monoclonal antibody against ErbB2, trastuzumab, and a tyrosine kinase inhibitor against EGFR, gefitinib, have improved the survival of breast and lung cancer patients. Unfortunately, cancer patients frequently become resistant to these inhibitors. This has led to investigating how EGFR can contribute to cell survival and how cancer cells can overcome inhibition of its signaling. Indeed, it is coming into focus that EGFR signaling goes beyond a single signal triggering cell proliferation and survival and is a sensor that regulates the cell’s response to microenvironmental stresses such as hypoxia. It acts as a switch that modulates the ability of cancer cells to survive. Autophagy is a process of self-digestion that is inhibited by EGFR allowing cancer cells to survive under stresses that would normally cause death and become resistant to chemotherapy. Inhibiting EGFR signaling allows autophagy to contribute to cell death. This gives new opportunities to develop novel therapeutic strategies to treat cancers that rely on EGFR signaling networks and autophagy. In this review, we summarize the current understanding of EGFR family member regulation of autophagy in cancer cells and how new therapeutic strategies could be developed to overcome drug resistance.

## 1. Introduction

The progression from normal cell to neoplastic disease is a multistep process where cells accumulate a variety of characteristics, now described as the hallmarks of cancer. These include the induction of angiogenesis, the activation of invasion and metastasis, the enabling of replicative immortality, the evasion of the immune system and growth suppressors, and changes in energy metabolism. The final hallmark is the resistance of cell death [1]. In this review, we will be examining the role of epidermal growth factor receptor (EGFR) family members in evading cell death, discussing how EGFR family member signaling can lead to cell survival through autophagy regulation and the opportunities for new therapies that exploit cancers that rely on EGFR family member signaling for their survival.

## 2. Epidermal Growth Factor Receptor Family

The EGFR family refers to the ErbB family of transmembrane receptor tyrosine kinases including EGFR/ErbB1/HER1, HER2/ErbB2/c-neu, HER3/ErbB3, and HER4/ErbB4 [2,3]. The ligands for these receptors include epidermal growth factor (EGF), heregulin, transforming growth factor alpha (TNFα), and heparin binding EGF like growth factor (HBEGF) [4]. Upon ligation of these ligands, the receptors form homo- and hetero-dimers [5,6]. This is required for activation of their kinase domains by transphosphorylation of the C-terminal domain [4]. This in turn recruits numerous binding proteins leading to the activation of several signaling pathways. These pathways activate downstream signaling cascades that lead to cell survival through a number of different mechanisms [7].

EGFR family members are involved in the progression of various types of cancers [3,8]. The overexpression of EGFR family members, including EGFR, ErbB2, ErbB3, and ErbB4, and/or mutation in these receptors can lead to more aggressive disease and resistance to chemotherapy [8]. In breast cancer, EGFR or ErbB2 are overexpressed. ErbB2 overexpression defines a subtype of breast cancer (HER2+ subtype). ErbB2 overexpression causes homodimerization and activation of its kinase independent of ligand [9]. In lung cancer, EGFR is also overexpressed, and in a subset of lung cancers EGFR is mutated, rendering the receptor constitutively activated [10]. In glioblastoma, EGFR is overexpressed or mutated which causes truncation of EGFR (EGFRvIII) and its constitutive activation [11]. These alterations are associated with more aggressive disease and resistance to therapy [2]. This resistance provides cells with mechanisms to bypass conventional chemotherapeutic targets.

Since EGFR family members are altered in cancers, targeted chemotherapy has been developed to inhibit activation of EGFR family members. The first of such a drug developed was an antibody against ErbB2 called trastuzumab, which binds and endocytoses the receptor, leading to its degradation and induction of apoptosis [12,13]. In addition, trastuzumab blocks cancer cells in the G1 phase of the cell cycle independent of endocytic downregulation [14]. In breast cancer, trastuzumab has extended survival in patients with overexpression of ErbB2 [3]. Unfortunately, breast cancer cells have developed drug resistance through shedding ErbB2 away from the targeting by trastuzumab, the truncation of ErbB2, or alterations in the downstream signaling pathways [15]. Expression prolife of the EGFR family members changes where ErbB3 is upregulated, replacing the ErbB2 function [16]. Other tyrosine kinase receptors such as MET also become upregulated rendering trastuzumab treatment ineffective [17]. To overcome these limitations, a small molecule inhibitor lapatinib was developed to inhibit both EGFR and ErbB2 kinase activity [18]. This has been effective, but breast cancer cells have frequently developed new drug resistance. A new drug resistance mechanism is the upregulation of Bcl-2 family members in an EGFR-independent manner [19,20,21]. Mutation in the binding pocket in EGFR for lapatinib but retaining in EGFR kinase activity have also been found [22]. Another antibody against ErbB2, pertuzumab, has been developed to treat HER2+ breast tumor in combination with trastuzumab [23]. This improved overall survival in patients with HER2+ breast cancer by increasing cell-mediated cytotoxicity (ADCC) to overcome resistance to trastuzumab [24]. To antagonize EGFR overexpressing cancers, two inhibitors against its kinase activity, erlotinib and gefitinib, have been developed [25]. This has been effective in treating lung cancers and colon cancer patients [26]. However, mutations such as T790M change make cancer cells become resistant to these drugs [27]. Antibody-based therapies against EGFR (cetuximab and panitimumab) are also used to treat lung and colon cancer patients. This allows for ADCC effects to occur [28]. Due to toxicities and drug resistance, these therapies have limited success, emphasizing the need to understand the interplay between cell survival and cell death pathways.

## 3. How EGFR Family Members Regulate Cell Survival

**Figure 1:**
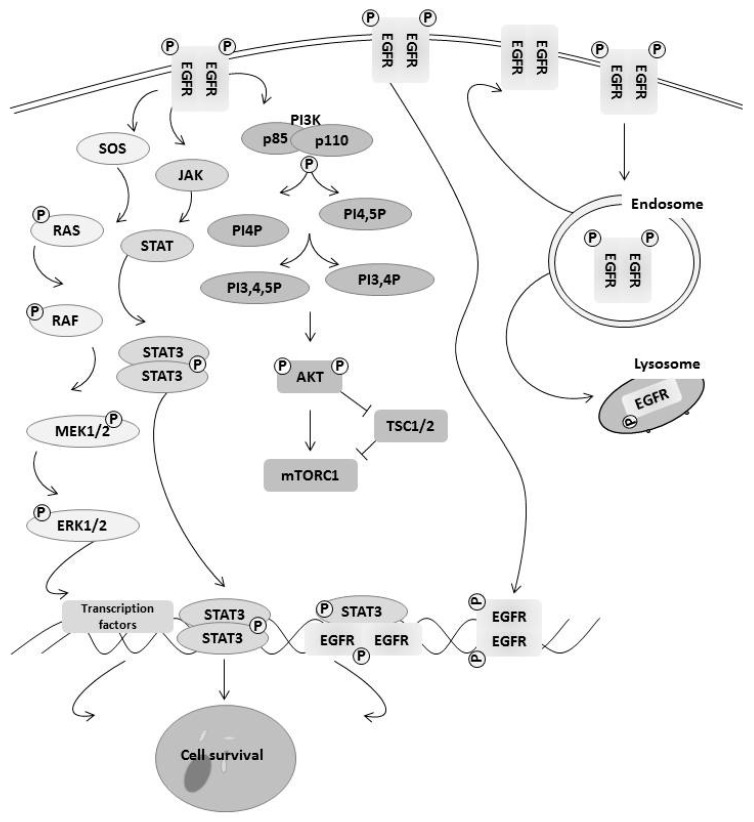
EGFR family members contribute to cell survival through multiple mechanisms. EGFR family members activate three of the major signaling pathways for cell survival including the RAS/MAPK pathway, the JAK/STAT pathway, and the PI3K/AKT pathway. Downstream signaling increases transcription levels of survival proteins, including Mcl-1 and Elk-1. It has also been shown that EGFR can translocate directly to the nucleus and activate transcription of survival genes. Finally, equilibrium between recycling EGFR back to the cell surface and sending it to the lysosome for degradation balances continued signaling.

EGFR family members contribute to cancer cell survival through multiple mechanisms including changes in signaling pathways, decreased degradation of the receptor through changes in the endocytosis pathway, and epigenetic changes that lead to changes in transcription (Figure 1). One of the major signaling pathways activated by EGFR family members is the Ras/MAPK pathway leading to increased cell growth and survival [29,30]. Mitogen-activated protein kinase (MAPK) pathways involve a three-layer system of proteins that are phosphorylated and then activated in a signaling cascade [29]. The most important of these MAPK pathways in cancer is the ERK pathway, which has been extensively studied. Briefly, when a receptor tyrosine kinase (RTK) is activated by its ligand, the receptors dimerize, autophosphorylate, and then form binding sites for either SH3 or phosphotyrosine domains within adaptor proteins. This leads to the transfer of son of sevenless (SOS) from the cytosol to the plasma membrane where it activates RAS, which activates RAF. RAF in turn activates MEK1/2 and ERK1/2 through phosphorylation of their kinase domains. This leads to activation of many transcription factors such as Elk1, which increased pro-survival genes [31]. We and others have shown that EGF activation of the RAS/ERK pathway leads to increased expression of anti-apoptotic protein Mcl-1 through Elk1 blocking apoptosis [32]. ERK1/2 proteins have been shown to directly phosphorylate EGF receptors as well as activate tyrosine phosphatases that can inactivate EGFR receptors in a negative feedback loop [33]. Another pathway activated by EGFR family members is the AKT signaling pathway. Phosphoinositide 3-kinase (PI3K) are lipid kinases where there are three main classes, but class 1 is the most important in tumorigenesis. Class 1 consists of two subunits, p110 which is the catalytic subunit, and the regulatory subunit p85. Phosphorylation of EGF receptors leads binding of p85, which then releases p110 through adaptor proteins such as Shc binding to the receptor. Upon release, p110 will phosphorylate phosphatidylinositol-3,4 biphosphate (PIP2) into phosphatidylinositol-3,4,5 biphosphate (PIP3) [34]. AKT is then recruited to the plasma membrane by PIP3 and phosphorylated via PDK1 at Thr308 and Ser473. AKT is a central signaling molecule, leading to inactivation of pro-cell death transcription factor FoxO family members and Bcl-2 family member Bad. It also activates NFkB transcription factor, leading to upregulation of pro-survival genes such as Bcl-2 family member Mcl-1 and Bcl-xL [35]. AKT also activates the mTOR pathway that regulates protein translation and contributes to cell survival [35]. Another major signaling pathway activated by EGFR family members is the JAK/STAT pathway. Similar to other signaling pathways, upon activation of the receptor, JAK1–3 become activated, leading the tyrosine phosphorylation of STAT3/5 and translocation to the nucleus where STAT3/5 participate in the upregulation of pro-survival genes including Bcl-2 family members [36]. Activation of these pathways contributes to cell survival from microenvironment stress or cancer treatments.

EGFR family members contributes to cell survival independent of its signaling. Endocytosis of the EGFR enables the receptor to recycle to cell surface without its ligand [37]. It could also be targeted to degradation by fusing endosome with lysosomes [37]. This regulates the level of activation of EGF receptor family members. It has been shown that continuous signaling induces an apoptotic pathway, rendering the recycling of EGFR family members as a cell survival mechanism. There is, however, a small amount of EGFR that translocates to the nucleus and regulates epigenetic changes and gene expression such as cyclin D1, contributing to drug resistance [38]. In several cancers, the amount of EGFR with nuclear localization was increased, indicating its ability to promote cancer cells to survive [39]. This places EGFR family members at the nexus of regulating cell survival.

## 4. Autophagy Regulates Both Cell Survival and Death

**Figure 2:**
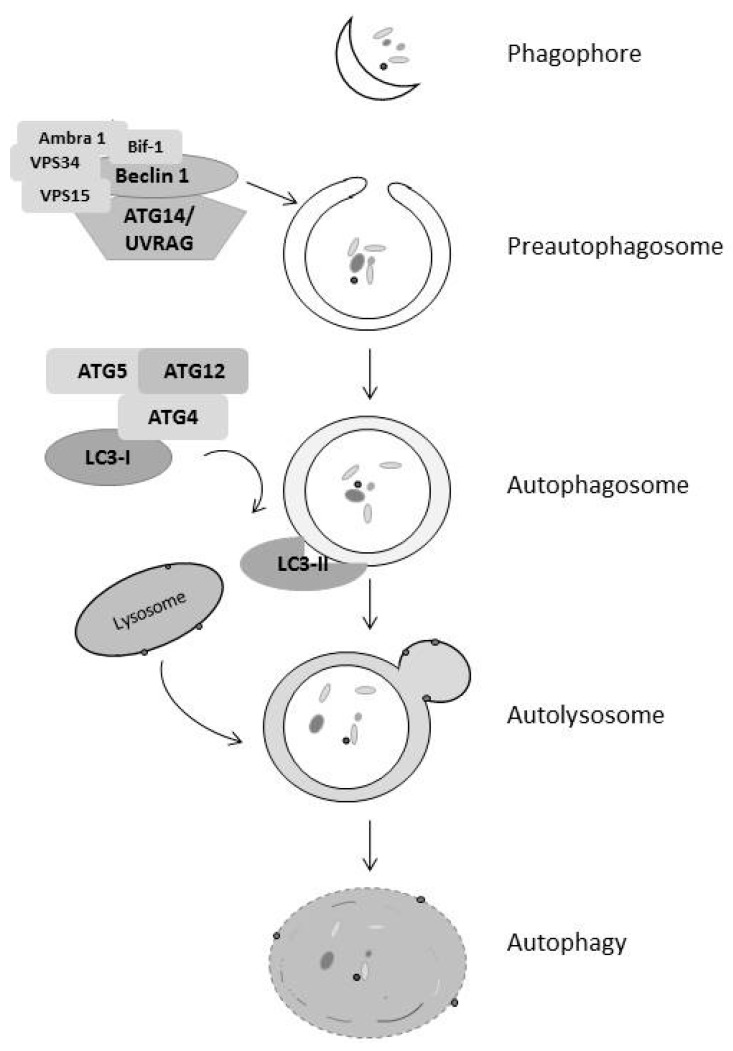
Stages of Autophagy. Upon induction of autophagy, the class III PI3K complex binds to beclin 1 forming the preautophagosome from the phagophore. Cytosolic LC3-1 is cleaved and lipidated to form LC3-II, which goes to the membrane of the autophagosome and leads to fusion of the autophagosome and the lysosome to form the autolysosome and subsequent breakdown of the vesicle and its contents.

Autophagy is a double-edged sword contributing to both cell survival and death. Autophagy is an important mechanism of cellular survival, through the recycling of intracellular components. It is a catabolic process involving lysosomes [40]. Based on how the intracellular components are delivered to lysosome for degradation, autophagy can be classified into three major types: macroautophagy, microautophagy, and chaperone-mediated autophagy [41]. Among these three types of autophagy, macroautophagy is the type that has received the most comprehensive and intensive studies. In this review, we will focus on this type of autophagy and, hereafter, autophagy refers to macroautophagy. During autophagy, intracellular cargoes including unfolded proteins, damaged organelles, and other materials are enclosed in a characteristic double-membraned structure called autophagosome. This is formed by activation of the class III PI3 kinase complex containing beclin-1, which generates a preautophagosome structure. These preautophagosome membranes acquire the Atg5-Atg12 complex and Atg8-phosphoethanolamine (PE) conjugate (LC3-I). The Atg4 protease cleaves LC3-I at the C-terminus to facilitate its lipidation (LC3-II), leading to autophagosome [42]. Autophagosome then fuses with lysosome to form autolysosome where intracellular cargoes are degraded and recycled back into the cytosol (Figure 2).

Autophagy can become over-activated so that the essential components to maintain cell survival are degraded. This can promote cell death, which is called autophagic cell death [42]. Furthermore, a subtype of autophagic cell death, called autosis, was recently characterized. It features focal concavity of the nuclear surface, focal ballooning of perinuclear space, and dilated and fragmented endoplasmic reticulum [43]. The regulation of autophagy will be critical if autophapgy contributes to cell survival or cell death.

## 5. Autophagy and Cancer

The contribution of autophagy to the development and progression of cancers is complex and controversial. Autophagy levels are often activity restricted in many cancers. For example, the autophagic gene beclin-1 is monoallelically deleted in human breast, ovarian, and prostate cancers [44]. It was found that beclin-1 expression is decreased in human breast cancers compared to normal breast tissue leading to reduced autophagy [45]. Gene knockout studies in mice indicate that *beclin1/atg6* functions as a tumor suppressor [46,47]. Mice lacking Atg4c are susceptible to fibrosarcomas [48]. In addition, in many cancers driven by growth factor signaling, mTOR activation is increased, thereby further restricting autophagy by inhibiting the ULK1 complex [49]. Conversely, autophagy plays important roles in protecting cancer cells from genotoxic and metabolic stress, leading to tumorgenesis [42]. In addition, autophagy degrades damaged or aggregated proteins and damaged mitochondria, which also contributes to tumorgenesis. Indeed, we and others have shown that, under hypoxia and starvation conditions, autophagy has a protective role at least in the short term [43,50]. Autophagy in the microenvironment may also limit the immune system infiltration of the tumor, allowing tumors to escape immune surveillance [51]. Autophagy in the tumor stromal cells recycles the damaged mitochondria and proteins to provide essential nutrients and energy for neighboring cancer cells, furthering promoting tumor progression and metastasis [52]. This illustrates the context of autophagy induction in cancer and will define its role in cancer progression and in how to target it for therapy.

## 6. EGFR Family Members Regulates Autophagy

**Figure 3:**
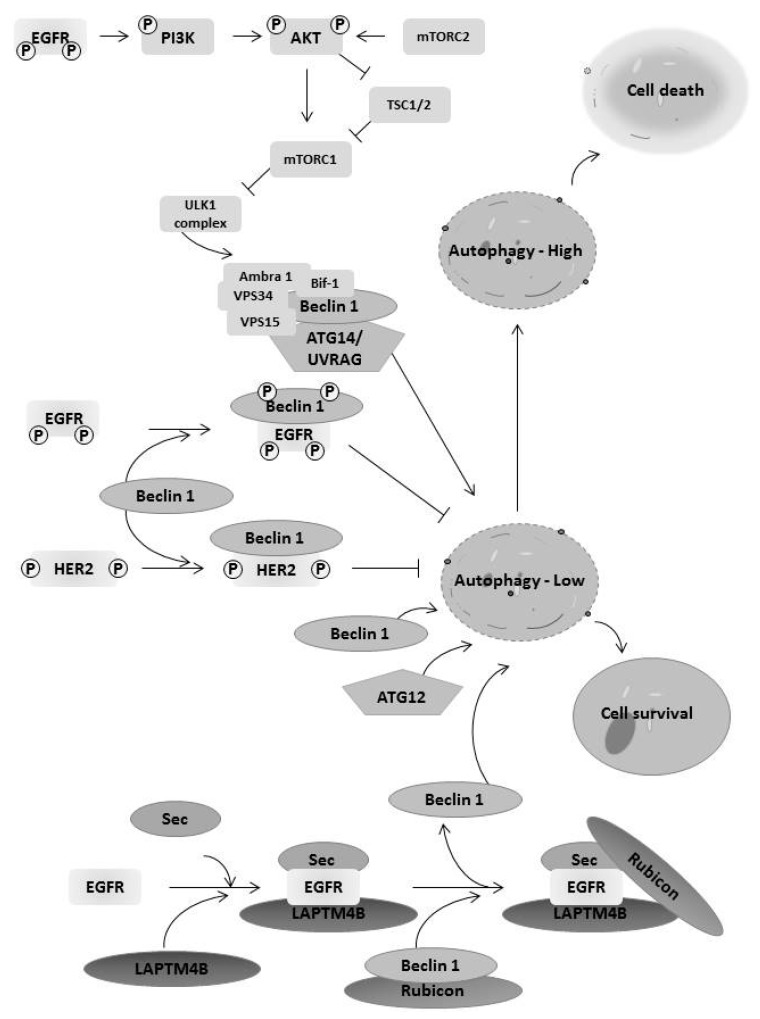
EGFR family members regulate autophagy affecting cancer cell survival and death. EGF receptor family members interact with key proteins in the autophagic pathway, leading to both cell survival and cell death dependent on the context. This includes activation of the mTOR pathway, leading to inhibition of the ULK1 complex, the binding of EGFR family members to beclin-1, and EGFR binding to LAPTM4B, releasing beclin-1 from Rubicon. The context of these interactions determines the level of autophagy, which is often dysregulated in cancer.

EGFR family members regulate autophagy affecting cancer cell survival and death. Activation of EGFR tyrosine kinase can inhibit autophagy [2,53,54]. EGFR activation leads to the inhibition of autophagy by the binding of EGFR to autophagy protein Beclin 1 and further reducing the Beclin 1 associated VPS34 kinase activity [54]. Another mechanism is to regulate expression of an autophagy protein by EGFR. The EGFR inhibition by the antibody cetuximab promotes autophagy by increasing expression of the autophagy protein Beclin 1. Cetuximab treatment suppresses the microRNA miR-216b that targets Beclin 1 mRNA to inhibit its translation [55]. In addition, EGFR upregulation of Bcl-2 binding to beclin-1 also limited autophagy induction [56]. EGFR also activates the AKT signaling pathway, causing phosphorylates TSC1 and thus leading to mTOR activation. This inhibits autophagy through inhibition of the ULK1 complex (Figure 3). The mTOR pathway also increases the translation of genes that might impact the induction of autophagy [57]. In contrast, EGFR was reported to regulate autophagy independently of its tyrosine kinase activity [58]. Inactive EGFR interacts with the oncoprotein LAPTM4B to form a subcomplex containing Sec5. The recruitment of the oncoprotein lysosomal-associated transmembrane protein 4B (LAPTM4B) and exocyst component Sec5 enhances the association of EGFR with the autophagy inhibitor Rubicon (RUN domain protein as Beclin 1-interacting and cysteine-rich containing), which releases Beclin 1 from Rubicon to initiate basal or serum starvation induced autophagy (Figure 3) [58]. Thus, EGFR seems to regulate both basal and inducible autophagy in a context-dependent manner.

EGFR is a target for therapy in many cancers. EGFR tyrosine kinase inhibitors (EGFR-TKI) induce autophagy and, in most cases, play a protective role in cancer cells (Table 1). Autophagy plays a pro-cell survival role in head and neck squamous cell carcinomas treated with erlotinib [59], in colorectal cancer cells treated with cetuximab [55,60], and in ovarian cancer cells treated with AG1478, another EGFR inhibitor [61]. Resistance of non-small cell lung cancer cells to treatment with gefitinib or erlotinib can be overcome by inhibition of autophagy [62,63]. However, when autophagy is further elevated by a treatment in addition to an EGFR-TKI, it can induce autophagic cell death. In erlotinib-resistant HeLa-R30 cells, the addition of rapamycin increased autophagy and cell death induced by erlotinib, and cell death induced by the combination of rapamycin and erlotinib was inhibited by knockdown of autophagy gene *ATG7* [64]. Similarly, in EGFR-TKI-resistant lung cancer cells with T790M mutation, the combination of a protein kinase CK2 inhibitor and an EGFR-TKI induced a high level of autophagy that degraded EGFR protein and promoted apoptosis [65]. The plant-extracted compound β-elemene can also enhance autophagy and cell death in gefitinib-treated glioblastoma cells [66]. Our recent study demonstrated that EGFR-modulated autophagy under hypoxia plays a dual role in cell survival and cell death in the same cancer cell line [50]. When cancer cell lines U87 and A549 were treated under hypoxia, the activity of EGFR tyrosine kinase decreased with prolonged treatment, which led to an increase in autophagy flux. The low level of autophagy in an early time of hypoxia protected cells from death, whereas the elevated level of autophagy in a later time of hypoxia promoted autosis [50]. Furthermore, the pro-cell survival and pro-cell death roles of autophagy can be switched by adding an EGFR-TKI at an early time of hypoxia or by re-activating EGFR at a later time of hypoxia. Our finding is in agreement with the report that tumors highly expressing EGFR have a low level of autophagic flux and highly rely on autophagy for survival and growth [67]. Autophagy induced by the tyrosine kinase-independent EGFR also supports cancer cell survival, which provides a mechanism for cancer cells to escape toxicity from EGFR-TKIs [58]. Another function of the tyrosine kinase-independent EGFR is to protect cancer cells from autophagic cell death induced by glucose starvation, when EGFR interacts and stabilizes the sodium/glucose cotransporter 1 (SGLT1) to maintain intracellular glucose level [68]. This suggests that EGFR target therapy is restricted by the context of EGFR regulation of autophagy.

The functional interplay between EGFR and autophagy is bidirectional where autophagy can regulate EGFR functions. Induction of autophagy triggered the localization of EGFR to mitochondria contributing to cell survival [69]. Autophagic degradation of EGFR was caused by the treatment of cancer cells with the protein kinase CK2 inhibitor CX-4945 [65] or with the herbal plant derivative celastraol [70]. This suggests that the induction of autophagy can limit EGFR survival signaling.

In addition to the regulation of autophagy by EGFR, other EGFR family members can also regulate autophagy. Similar to EGFR, ErbB2 inhibits autophagy via the formation of a complex with Beclin 1 [71]. ErbB2 also regulates autophagy through other mechanisms. In different subtypes of patient breast tumor tissues, tissue expressing high ErbB2 have less expression of the autophagy proteins LC3A, LC3B, and Beclin 1 compared to tissues expressing low ErbB2 [72]. When ErbB2-overexpressing breast cancer cells were treated with the trastuzumab, the autophagy protein ATG12 was differentially upregulated [73]. In contrast, a recent study reported that autophagy protein ATG4B has a positive association with ErbB2 in a subtype of breast cancer cells [74]. It is expected that ErbB2-regulated autophagy plays a role in cell survival and death. Autophagy protected ErbB2 overexpressing breast cancer cells from trastuzumab cytotoxicity [73,75]. Lapatinib, a dual inhibitor of EGFR and ErbB2 tyrosine kinases, induced autophagic cell death in ErbB2 overexpressing breast cancer cells [76], human hepatoma cells [77], and acute myeloblastic leukemia [78]. On the other hand, ErbB2 degradation by autophagy in breast cancer cells was recently reported requiring polyubiquitinationin [79,80]. The roles of ErbB3 and ErbB4 in autophagy await investigation. One study showed that p38α inhibition leads to FoxO3A activation, autophagy, and FOxO3A-dependent ErbB3 upregulation [81]. However, the role of ErbB3 in autophagy is unknown. The tumor suppressor WWOX (WW domain-containing oxidoreductase) suppresses autophagy in human squamous cell carcinoma [82]. Furthermore, WWOX was shown to interact with ErbB4 [83]. Thus, EGFR and ErbB2 regulate autophagy that contributes to drug resistance. The roles of ErbB3 and ErbB4 in regulating autophagy remains unclear.

## 7. Conclusions

EGFR family members restrict autophagy during cancer progression through signaling and receptor complexes with autophagy genes. In contact, target therapy toward EGFR family members induce autophagy contributing to cell survival. The complexing roles of autophagy in cancer may provide therapeutic opportunities to target autophagy in context to EGFR family members. Basal autophagy is critical for maintaining hemostasis in cancer cells but must be limited to prevent intracellular collapse. If this limitation is driven by EGFR family members, it would make sense to inhibit EGFR in combination with autophagy inducers leading cells to autosis. In cancers where EGFR family members are not drivers of cancer progression, it would be advantageous to inhibit autophagy in combination with targeted therapy to other cancer drivers. Understanding these interactions of growth factor signaling (EGFR) and stress responses (autophagy) will provide more effective therapeutic strategies to the cancer characteristics and hence the patient.

## Figures and Tables

**Table 1:** EGFR tyrosine kinase inhibitors (EGFR-TKI) induce autophagy and, in most cases, play a protective role in cancer cells.

Therapy	Target	Autophagy	Disease Site
lapatinib	EGFR/ErbB2	Induction	breast cancer
rapamycin	mTOR	Induction	renal cancer
cetuximab	EGFR	Induction	colon cancer/head and neck cancer
trastuzumab	ErbB2	Induction	gastric cancer/breast cancer
neratinib	EGFR/ErbB2	Unknown	Not FDA approved
afatinib (BIBW2992)	ErbB2	Unknown	lung cancer
pertuzumab	ErbB2	Unknown	breast cancer
gefitinib	EGFR	Induction	lung cancer
panitumumab	EGFR	Induction	colon cancer
erlotinib	EGFR	Induction	lung and colon cancer
AG1478	EGFR	Inhibition	ovarian
β-elemene	ATG-5	Induction	gastric cancer
CX-4945	Casein Kinase 2	Induction	Not FDA approved
